# Evaluating Avascular Necrosis-Related Content Across Social Media Platforms

**DOI:** 10.7759/cureus.87914

**Published:** 2025-07-14

**Authors:** Gabriella B Smith, Anna V Gussner, Grady H Hofmann, Nneoma O Duru, Kevin G Shea

**Affiliations:** 1 Orthopaedic Surgery, Stanford University School of Medicine, Stanford, USA

**Keywords:** avascular necrosis, avn, orthopaedics, osteonecrosis, social media, social media content

## Abstract

Background

Avascular necrosis (AVN) of the bone can progress to subchondral collapse, often requiring surgical intervention. Given the debilitating nature of this condition, patients may seek information and support through social media. AVN-related content on social media platforms remains uncharacterized in the literature. This study aimed to analyze the type and reach of AVN-related content across social media platforms.

Methods

The search terms “avascular necrosis” and “osteonecrosis” were used to identify social media content on Facebook, Instagram, YouTube, and TikTok between October 31, 2024, and November 4, 2024. Posts with the highest engagement were reviewed, and descriptive statistics were recorded.

Results

Across all four platforms, engagement metrics for “avascular necrosis” were consistently higher than for “osteonecrosis.” The following findings were noted for the search term “avascular necrosis.” On Facebook, five groups with a combined membership of 30,805 shared 492 posts monthly. Three groups provided patient support, while the remaining two focused on a patient story and treatment advice; only one group had a physician moderator. Instagram’s top five hashtags had over 24,000 posts. On YouTube, the five most-viewed videos - with 1.78 million combined views - addressed topics such as patient experiences, surgical techniques, and education. TikTok had 135 videos for “avascular necrosis,” with the most popular video receiving 5.6 million views and 163,200 likes. The top five TikTok hashtags had a total of 2,218 posts.

Conclusion

AVN-related content is widely available on social media platforms, offering patients access to support networks and informational resources. However, concerns regarding the reliability and accuracy of educational content persist. Physicians treating AVN patients should familiarize themselves with this digital landscape to address patient misconceptions and share credible sources. Future educational initiatives could involve creating physician-led social media content to enhance the accessibility and quality of AVN-related resources.

## Introduction

Avascular necrosis (AVN), also known as osteonecrosis, is a degenerative condition resulting from the loss of subchondral blood supply, most commonly to the femoral head [1]. The disruption of the subchondral blood supply is associated with various etiologies, including corticosteroid use, alcohol abuse, and radiation therapy [2]. If AVN is left untreated, it can lead to progressive bone destruction and joint collapse, ultimately necessitating surgical intervention, such as joint replacement [3]. Treatment options vary depending on the underlying cause and the progression of the disease [4]. The multifactorial etiology and range of treatment options - depending on patient factors and time of presentation - can make understanding AVN challenging for patients. 

Social media provides a unique, interactive platform where individuals, including patients, can exchange information, access educational resources, and share personal experiences [5]. Platforms such as Facebook, Instagram, YouTube, and TikTok facilitate forums for education, peer support, and patient storytelling [6-9]. The quality and accuracy of health-related content on these platforms can vary significantly [10], which may influence patient decision-making and behaviors [11]. Previous studies have evaluated social media content for other medical conditions, highlighting both the benefits and risks of using social media as a health information source [8,9,12].

Despite the significant impact of AVN and its associated treatment on patient quality of life, AVN-specific content on social media has not been systematically evaluated [13]. Characterizing the social media health information landscape for AVN is critical for helping providers understand patients’ knowledge, support shared decision-making, and guide effective patient education and support interventions. The purpose of this study is to determine the reach and engagement of AVN-related information on social media, to assess whether social media is a potential avenue for patient education efforts and for addressing misinformation related to AVN.

## Materials and methods

Between October 31, 2024, and November 4, 2024, Facebook, Instagram, YouTube, and TikTok were queried separately using the search terms “avascular necrosis” and “osteonecrosis.” This study was exempt from IRB approval.

Measurable metrics for publicly accessible content on four social media platforms were recorded separately for each search term. For Facebook, the five groups with the largest memberships were identified, and data on membership count, post frequency, thematic focus, and the presence of a physician moderator were collected. For Instagram, the top five hashtags were identified for each search term based on post count and relevance. On YouTube, the five most-viewed videos for each search term were analyzed; data collected included view count, content theme, and whether a physician posted the video. For TikTok, the top five hashtags for each search term were identified based on post count. The five most-viewed TikTok videos for each search term were also analyzed, with likes, views, content category, and whether a physician posted the video recorded.

Engagement metrics such as views, posts, likes, and group members were analyzed across all platforms. These metrics were chosen as proxies for content engagement on each social media platform, reflecting the frequency of content shared, the volume of content, and the reach of that content. Physician involvement was determined on Facebook, YouTube, and TikTok by checking whether the posting account explicitly listed “MD” or “DO” in their profile. Content was categorized into the following themes: educational, patient stories, case studies, support groups, treatment guidance, physician self-promotion, or surgical techniques.

## Results

For the search term “avascular necrosis,” the top five Facebook groups were identified, with 30,805 members collectively. The largest group, Avascular Necrosis Support Group, had 11,811 members and 432 posts per month (Table 1). Thematic analysis revealed that three groups focused on patient support, one highlighted a patient’s story, and another provided treatment guidance. Physician involvement was noted in only one of these groups.

**Table 1:** Top Facebook Groups Search Term "Avascular Necrosis"

Name of Facebook Group	Type of Group	Total Membership	Posts Per Month	Physician Moderator
Avascular Necrosis Support Group	Support Group	11,811	432	Yes
Avascular Necrosis (AVN) Non-Operative Treatment Group	Treatment Guidance	3,717	34	No
Avascular Necrosis Support Est. 2012	Support Group	4,186	5	No
Avascular Necrosis Young Support Group	Support Group	1,766	3	No
Matthew's LIFE After Leukemia (4x), TMA, Stroke, & Avascular Necrosis	Patient Story Group	9,325	20	No

For the term “osteonecrosis,” the top five groups on Facebook had a total of 5,549 members and 44 posts per month. The top group, named Osteonecrosis, included 1,794 members and four posts per month (Table 2). Four of the groups centered on patient support, while one was dedicated to physician self-promotion. Physician involvement was similarly limited to one group. 

**Table 2:** Top Facebook Groups Search Term "Osteonecrosis"

Name of Facebook Group	Type of Group	Total Membership	Posts Per Month	Physician Moderator
Osteonecrosis	Support Group	1,794	4	No
Avascular Necrosis/Osteonecrosis Support Int'l.	Support Group	1,617	6	No
Avascular Necrosis and Osteonecrosis Treatment	Physician Self-Promotion	850	0	Yes
Living With Osteonecrosis of the Jaw	Support Group	943	28	No
Osteonecrosis	Support Group	345	6	No

The top five Instagram hashtags for “avascular necrosis” were #avascularnecrosis, #avascularnecrosisofthehips, #avascularnecrosissucks, #avascularnecrosissurvivor, and #avascularnecrosisofthefemoralhead. The most popular hashtag, #avascularnecrosis, had over 24,000 posts (Table 3). The corresponding top hashtag for the search term “osteonecrosis” was #osteonecrosis, with over 5,000 associated posts (Table 4). Posts under these hashtags primarily focused on personal stories of living with AVN. 

**Table 3:** Most Popular Avascular Necrosis (AVN)-Related Hashtags on Instagram and TikTok Search Term "Avascular Necrosis"

Instagram Hashtag	Number of Posts
#avascularnecrosis	22.4k
#avascularnecrosisofthehips	1000+
#avascularnecrosissucks	100+ (294)
#avascularnecrosissurvivor	100+ (231)
#avascularnecrosisofthefemoralhead	100+ (164)
TikTok Hashtag	Number of Posts
#avascularnecrosis	2,196
#avasculornecrosis	3
#avascularnnecrosis	1
#avascular_necrosis	3
#avascularnecrosisnigeria	15

**Table 4:** Most Popular Avascular Necrosis (AVN)-Related Hashtags on Instagram and TikTok Search Term "Osteonecrosis"

Instagram Hashtag	Number of Posts
#osteonecrosis	5000+
#osteonecrosisofthejaw	100+ (112)
#osteonecrosisfemoral	Fewer than 100 (17)
#osteonecrosisofthehip	Fewer than 100 (12)
#osteonecrosismaxilar	Fewer than 100 (37)
TikTok Hashtag	Number of Posts
#osteonecrosis	890
#osteonecrose	268
#osteonecrosiswarrior	4
#osteonecrosiss	1
#osteonecrosisbilateral	32

For the search term “avascular necrosis,” the five most-viewed YouTube videos had a combined total of 1.78 million views. The content themes included two videos featuring educational material, two focusing on patient stories, and one showcasing a surgical technique. The most-viewed video, titled “I Have Avascular Necrosis | Episode 63,” accumulated 908,000 views and focused on an individual patient’s journey. Four out of the top five videos were posted by physicians. For “osteonecrosis,” the five most-viewed YouTube videos had a total of 1.4 million views. Content themes included four videos providing educational material and one presenting a case study. Like the “avascular necrosis” results, four out of the top five videos were posted by physicians.

On TikTok, 135 videos corresponded to the search term “avascular necrosis.” The most popular video accumulated 5.6 million views and 163,000 likes. All five of the top videos featured patient stories, and two included an educational component posted by physicians. The top five hashtags were “#avascularnecrosis,” “#avasculornecrosis,” “#avascularnnecrosis,” “#avascular_necrosis,” and “#avascularnecrosisnigeria.” These hashtags collectively included 2,218 posts (Table 3).

For the search term “osteonecrosis” on TikTok, 146 videos were identified. The most popular video reached 472,000 views and 24,500 likes. Content themes included two videos featuring patient stories, two providing educational content, and one case study sharing surgical information. Physicians posted two of the top five videos. The top five hashtags had a combined total of 1,195 posts (Table 4). 

## Discussion

This study characterizes social media trends across four popular platforms and found that social media acts as a popular source of information and support for AVN patients. Despite several studies highlighting the role of social media in patient health education and decision-making, little is known about the social media landscape for AVN patients. Our findings may provide surgeons with an overview of the types of content that inform patient decision-making and AVN knowledge. Our findings highlight that physicians should consider utilizing social media to circulate accessible, high-quality AVN patient education resources. These findings may improve AVN patient-provider communication and AVN patient education resources. 

Across platforms, “avascular necrosis” consistently outperformed “osteonecrosis” in engagement, except on TikTok, where “osteonecrosis” had slightly more videos. However, metrics for the top “avascular necrosis” TikTok videos remained significantly higher, indicating greater overall engagement with this term. This discrepancy suggests that “avascular necrosis” may be more effective for clinicians developing educational materials.

Facebook emerged as a platform for patient support, with groups providing spaces for individuals to share experiences, seek advice, and build community [14,15]. However, limited physician involvement raises concerns about the accuracy and reliability of shared information. This finding aligns with previous research showing that online patient forums often lack professional oversight, which can contribute to the spread of anecdotal or unverified information [16]. 

Instagram and TikTok draw younger demographics, leveraging visual and engaging formats to share personal stories and experiences [17,18]. While these stories provide emotional support and relatable experiences for patients, personal narratives pose challenges when treatment options are specific to disease etiology and individual risk factors [19]. TikTok’s viral nature amplifies both the benefits and risks associated with rapid information dissemination, emphasizing the need for evidence-based material to mitigate misinformation.

Finally, YouTube emerged as the platform most focused on educational content, with healthcare professionals producing four of the top five videos for both search terms. This physician-generated content provided in-depth educational material, including explanations of surgical techniques and case studies, leveraging the platform’s long-form format to enhance patient understanding, as noted in prior studies [20].

These findings highlight both the opportunities and challenges presented by social media as a tool for AVN education and support. AVN predominantly affects a relatively young demographic, with a median age of onset of 12.1 [21], making social media platforms effective channels to reach these patients [22]. Beyond the physical burden, chronic pain, physical limitations, and decreased psychosocial well-being can impact patients’ quality of life and social functioning [23]. These engagement metrics suggest that social media can provide education, emotional support, and a sense of community to patients. 

Physicians treating AVN patients should be aware of the information patients encounter online and address potential misconceptions during clinical encounters. By guiding patients toward credible sources and encouraging shared decision-making, healthcare providers can foster trust, improve communication, and empower patients to make informed decisions about their care [24,25].

Similar orthopedic studies reveal social media’s dual role as a source of support and potential misinformation. Prior research groups’ study on pediatric ACL injuries highlights that social media platforms like Facebook and Instagram provide valuable emotional support for patients and caregivers; however, limited physician oversight may compromise the accuracy of the advice shared [9]. Studies on clubfoot and osteochondritis dissecans demonstrate social media’s value in promoting evidence-based treatments while fostering supportive communities [8,12]. These findings align with the current study’s observations, reinforcing the notion that physician engagement on social media is beneficial.

Social media use among physicians is on the rise [26]. In 2014, 90% of physicians reported using social media for personal activities, and 65% used it for professional reasons [27]. Platforms like YouTube, Instagram, and TikTok allow healthcare professionals to develop high-quality, engaging content tailored to patient needs [28]. Physician-led initiatives could include educational videos explaining AVN’s etiology, treatment options, progression, and patient stories, combined with expert guidance. 

This study is not without limitations. Engagement metrics, such as likes, views, and member counts, may not necessarily reflect patient satisfaction or real-world impact. The cross-sectional design of this study does not account for content changes over time, which may influence both reach and relevance. Additionally, given the dynamic nature of social media, these specific findings may not be replicable, as hashtags and post counts change over time. While we selected a narrow study window to ensure we were collecting across all platforms at the same time, we acknowledge that this limited our ability to assess trends or fluctuations over time. Social media platforms such as X and Snapchat were excluded due to limited metric reporting [15]. Private and closed groups were not included, potentially introducing bias into our sample. Content categorized as “physician involvement” relied on self-reported credentials, which may not always be verifiable, and we did not collect additional details on the types of physicians creating content in this study.

Future studies should incorporate methods to assess content quality, qualitative analysis of themes, and trends in social media usage for AVN over a longer study period. Public health interventions involving physician-developed content could improve patient education and decision-making. By incorporating social media into patient education strategies, healthcare professionals can better meet patients’ informational needs in a digital landscape.

## Conclusions

The impact of social media will continue to shape how patients access information and interact with providers. Platforms provide access to education and peer support; however, concerns about content accuracy and professional moderation highlight the opportunity for greater physician engagement in these spaces. Physicians can create and moderate accurate, engaging, and accessible content. By understanding the types of information patients encounter online, and incorporating this knowledge into shared decision-making discussions, healthcare providers can help ensure the dissemination of accurate, high-quality AVN information, ultimately improving patient education and care outcomes. Future efforts should focus on physician-led initiatives to improve the accuracy of social media content, and further research to evaluate its impact on patient care.
